# Quality of life after open descending thoracic and thoracoabdominal aortic repair: A prospective cohort study

**DOI:** 10.1016/j.xjon.2026.102081

**Published:** 2026-08-25

**Authors:** Pim S. van Sambeeck, Nesar A. Hasami, Stefan R. van Dinter, Foeke J.H. Nauta, Nabil Saouti, Michel W.A. Verkroost, Marieke Zegers, Guillaume S.C. Geuzebroek, Mark van den Boogaard, Robin H. Heijmen

**Affiliations:** a Department of Cardiothoracic Surgery, Radboud University Medical Center, Nijmegen, The Netherlands; b Department of Intensive Care, Radboud University Medical Center, Nijmegen, The Netherlands

**Keywords:** quality of life, thoracic aneurysm, thoracoabdominal aneurysm, open repair

## Abstract

**Objective:**

To longitudinally evaluate health-related quality of life (HRQoL) following open descending thoracic or thoracoabdominal aortic aneurysm repair, with validated patient-reported outcome measures.

**Methods:**

In this prospective, single-center cohort study, patients who underwent open descending thoracic or thoracoabdominal aortic aneurysm repair between 2016 and 2023 were included. HRQoL was assessed preoperatively and at 3, 12, and 24 months postoperatively with the Short Form-12 physical component summary (PCS) and mental component summary (MCS) scores. Secondary quality-of-life scores were included as well. Additionally, complete-case analysis and the impact of baseline variables were assessed. Longitudinal changes were analyzed with linear mixed-effects models.

**Results:**

In total, 128 patients were included. PCS scores (95% CIs) decreased from 46.0 (44.1-48.0) preoperatively to 39.7 (37.6-41.7) at 3 months, with partial recovery to 42.2 (40.2-44.2) at 1 year and 41.0 (38.1-43.9) at 2 years (overall *P* < .001). MCS scores (95% CIs) decreased initially from 47.4 (44.9-49.8) to 44.3 (41.8-46.9) (3 months), with recovery to 49.2 (46.7-51.7) (1 year) and 48.4 (44.6-52.2) (2 years) (overall *P* = .01). Complete-case analysis showed comparable trajectories. Anxiety decreased and cognitive complaints increased but remained within normal ranges. No baseline variables were independently associated with PCS or MCS score trajectories.

**Conclusions:**

Open descending thoracic and thoracoabdominal aortic aneurysm repair is associated with a sustained reduction in physical HRQoL, while mental HRQoL recovers. These findings underscore the multidimensional nature of recovery and the importance of integrating patient-reported outcomes into perioperative care.

## Graphical Abstract

**Figure:**
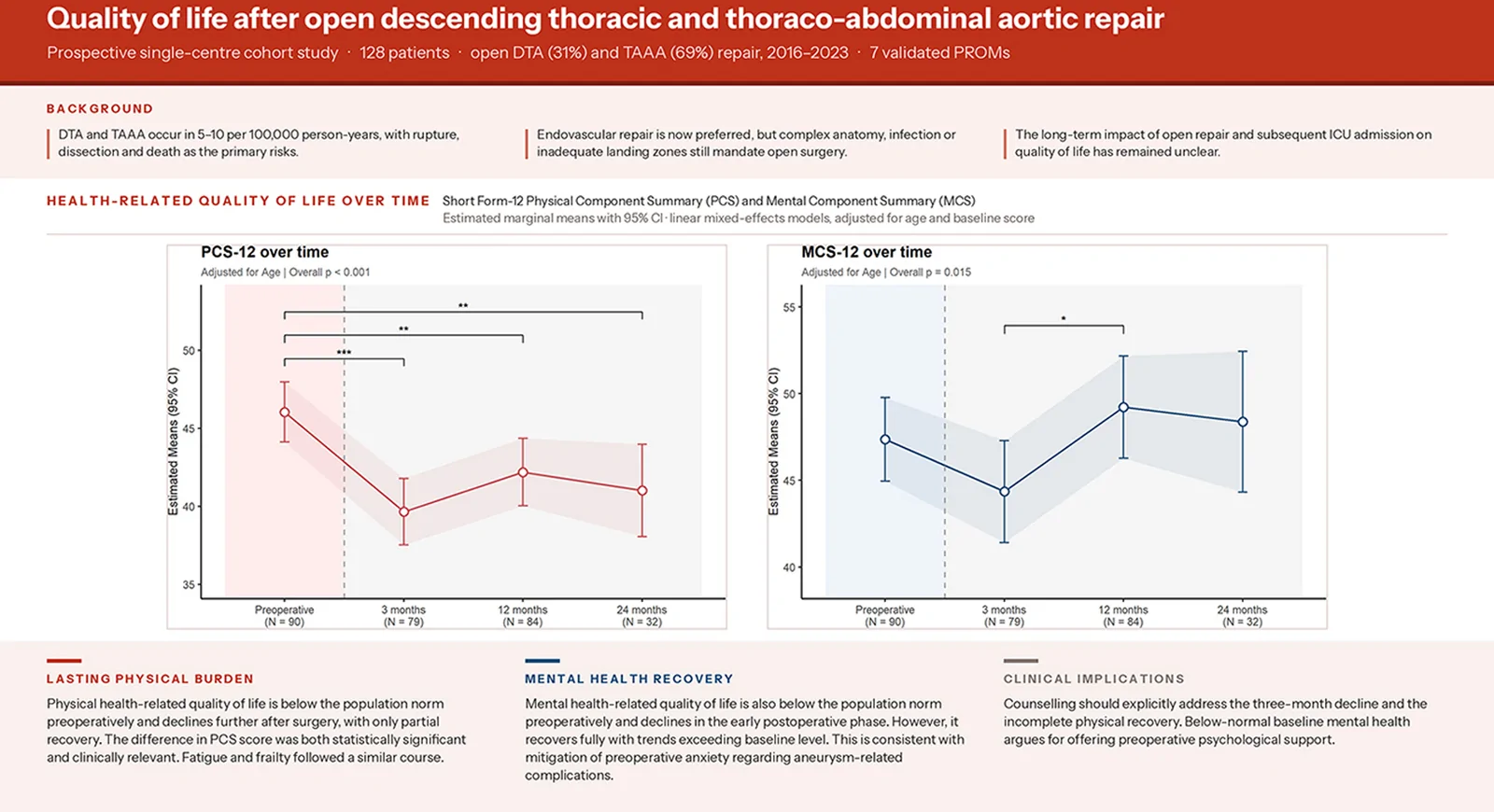

**Figure:**
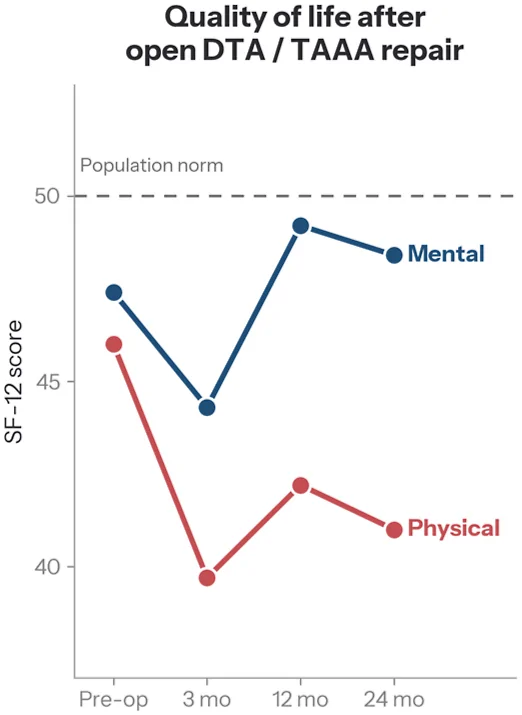
Quality-of-life trajectories after open descending thoracic and thoracoabdominal repair.

**Central Message**

After open descending thoracic and thoracoabdominal aortic repair, physical quality of life stays reduced, whereas mental quality of life recovers.

**Perspective**

These findings should prompt preoperative counseling before open descending thoracic and thoracoabdominal aortic repair: physical quality of life declines persistently, whereas mental quality of life recovers. Routine patient-reported outcome measurement may improve shared decision-making and perioperative care.

Descending thoracic and thoracoabdominal aortic aneurysms (DTA and TAAA) have an incidence estimated at 5 to 10 cases per 100,000 person-years.^1^ The primary risks are rupture, dissection, and death, with a substantially increased risk as aortic diameter exceeds 60 mm, which has prompted guideline recommendations for elective surgery at this threshold (or 55 mm in specific patient groups).^1^

Anatomical complexity, such as postdissection aneurysms, infection, or inadequate landing zones, may preclude the endovascular approach and indicate open surgical repair in selected cases.^2^ Perioperative morbidity, including cardiac complications, acute kidney injury, spinal cord ischemia, stroke, and multiorgan failure, remains an important risk,^3^, ^4^, ^5^ though outcomes are favorable in specialized aortic centers even for extensive TAAA repairs.^6^

Besides early postoperative outcomes, the long-term impact of open surgical repair and subsequent intensive care unit (ICU) admission on health-related quality of life (HRQoL) remains unclear.^7^ HRQoL encompasses physical, psychological, and social functioning and is typically assessed with validated patient-reported outcome measures (PROMs).^8^ Although previous studies generally report a decline in HRQoL after surgery, the evidence is limited by outdated cohorts, small sample sizes, heterogeneous designs, a primary focus on physical HRQoL, and a lack of comprehensive longitudinal preoperative and postoperative assessment.^5^^,^^9^ Therefore, this study aims to evaluate longitudinally HRQoL after open DTA and TAAA surgery with validated physical, mental, and cognitive PROMs.

## Methods

This study is a collaboration with the MONITOR-IC study (www.clinicaltrials.gov identifier NCT03246334),^10^ a multicenter prospective longitudinal cohort of Dutch ICU wards collecting multiple PROMs at multiple time points with validated questionnaires. Since only one participating center performs open DTA and TAAA repair, this analysis represents single-center data. The study was approved by the research ethics committee of the Radboud University Medical Center on August 23, 2016 (Arnhem-Nijmegen, The Netherlands) (2016-2724).

### Patient Eligibility and Enrolment

This study used convenience sampling. All adult patients who underwent open DTA or TAAA repair between 2016 and 2023 were eligible with no additional exclusion criteria. Patients planned for postoperative ICU admission were screened and, if eligible, given study information. Written informed consent was obtained preoperatively. In cases of emergency surgery, retrospective enrollment was performed.

### Surgical Technique

The surgical approach for open DTA and TAAA repair has been described previously.^2^^,^^6^ Patients undergo either a thoracotomy or thoraco-phreno-laparotomy, depending on the extent of aortic pathology. One-lung ventilation and distal aortic perfusion are employed in all cases, with conventional extracorporeal circulation, partial extracorporeal circulation, or left-heart bypass. Aortic clamping proceeds sequentially in a cranio-caudal fashion to allow Dacron graft implantation. Intercostal and lumbar arteries between T7 and L4 are reimplanted when feasible, visceral perfusion is established during clamping of the visceral segment, and nephroplegia is administered every 30 minutes when the kidneys are excluded. Patients at risk for medullary ischemia receive a prophylactic external lumbar drain preoperatively and are admitted to the medium care unit prior to surgery. Postoperatively, patients are transferred to the ICU.

### Data Collection

Patients completed questionnaires online or on paper, preoperatively in elective cases or retrospectively to recall preoperative status. Proxies assisted when patients were unable to complete questionnaires.^10^ Follow-up assessments were performed at 3 months, 1 year, and 2 years. Medical record data including demographics, medical history, aneurysm characteristics, surgical details, and ICU data were collected concurrently.

### Health-Related QoL Assessment

Multiple PROMs assessed physical, mental, and cognitive functioning (Table E1). The Short Form-12 yields a physical component summary (PCS) and mental component summary (MCS) score.^11^ The EuroQol 5-Dimension, 5-Level (EQ-5D-5L) captures health status via a utility index and visual analogue scale.^12^ Frailty and fatigue were measured with the Clinical Frailty Scale (CFS)^13^ and checklist individual strength (CIS-8).^14^ Anxiety and depression were assessed with the Hospital Anxiety and Depression Scale (HADS),^15^ posttraumatic stress with the Impact of Event Scale-6 (IES-6),^16^ and cognitive failure with the Cognitive Failure Questionnaire-14 (CFQ-14).^17^

### Outcomes

The primary outcomes were longitudinal changes in PCS-12 and MCS-12 scores over time, given their prevalence and comparability with previous literature.^5^ Secondary outcomes included changes in additional PROM scores, the association of baseline variables with PCS-12 and MCS-12 trajectories and calculation of the minimal clinically important difference (MCID) of the 12-item Short-Form Health Survey scores.

### Statistical Analysis

Statistical analyses were performed in R (R Foundation for Statistical Computing) using lme4, lmerTest, emmeans, and car. Model assumptions were evaluated prior to analysis, including linearity of continuous covariates, normality and homoscedasticity of residuals, and associations between dropout and missing data. Random slopes for time were included when they improved model fit.

Longitudinal changes in PCS-12, MCS-12, and secondary outcomes were analyzed using linear mixed-effects models with time as a categorical fixed effect and participant as a random intercept. Age and baseline score were included as covariates based on clinical relevance and their association with missing data. Overall time effects were assessed using Type II Wald χ^2^ tests. Exploratory associations between baseline covariates and longitudinal scores were examined using univariable and multivariable models adjusted for time, including clinically relevant covariates.

Subgroup analyses (DTA vs TAAA, elective vs acute presentation, uncomplicated vs complicated postoperative course) at each time point were analyzed using Welch *t*-tests or Mann-Whitney U tests depending on normality (Shapiro-Wilk). Post hoc comparisons were adjusted using Holm-Bonferroni correction. Missing items of PROMs were handled using the half-rule, replacing absent items with the mean of completed items, provided at least half of the (sub)scale items were answered. In the absence of anchor-based MCID values for aortic surgery, the MCID was calculated using the distributional method (0.5 standard deviation of baseline scores) and compared to cardiac surgery literature.^18^^,^^19^

## Results

### Patient Characteristics and Surgical Data

A total of 128 patients were included (Figure 1). Median age was 65.5 years (interquartile range, 57.3-70.8), 57% were male, and mean body mass index (BMI) was 26.8±4.4 kg/m^2^. Most procedures were elective (89.8%), and nearly all patients were in a stable preoperative condition (99.2%). A total of 88 (68.8%) of the participants were treated for a TAAA and 40 (31.2%) for a DTA. According to the Crawford classification, type I aneurysms predominated (34.1%). Common comorbidities included coronary artery disease (14.8%), pulmonary disease (18.0%), and peripheral artery disease (20.3%), while previous aortic surgery had been performed in 52.3% of patients (Table 1).

**Figure 1:**
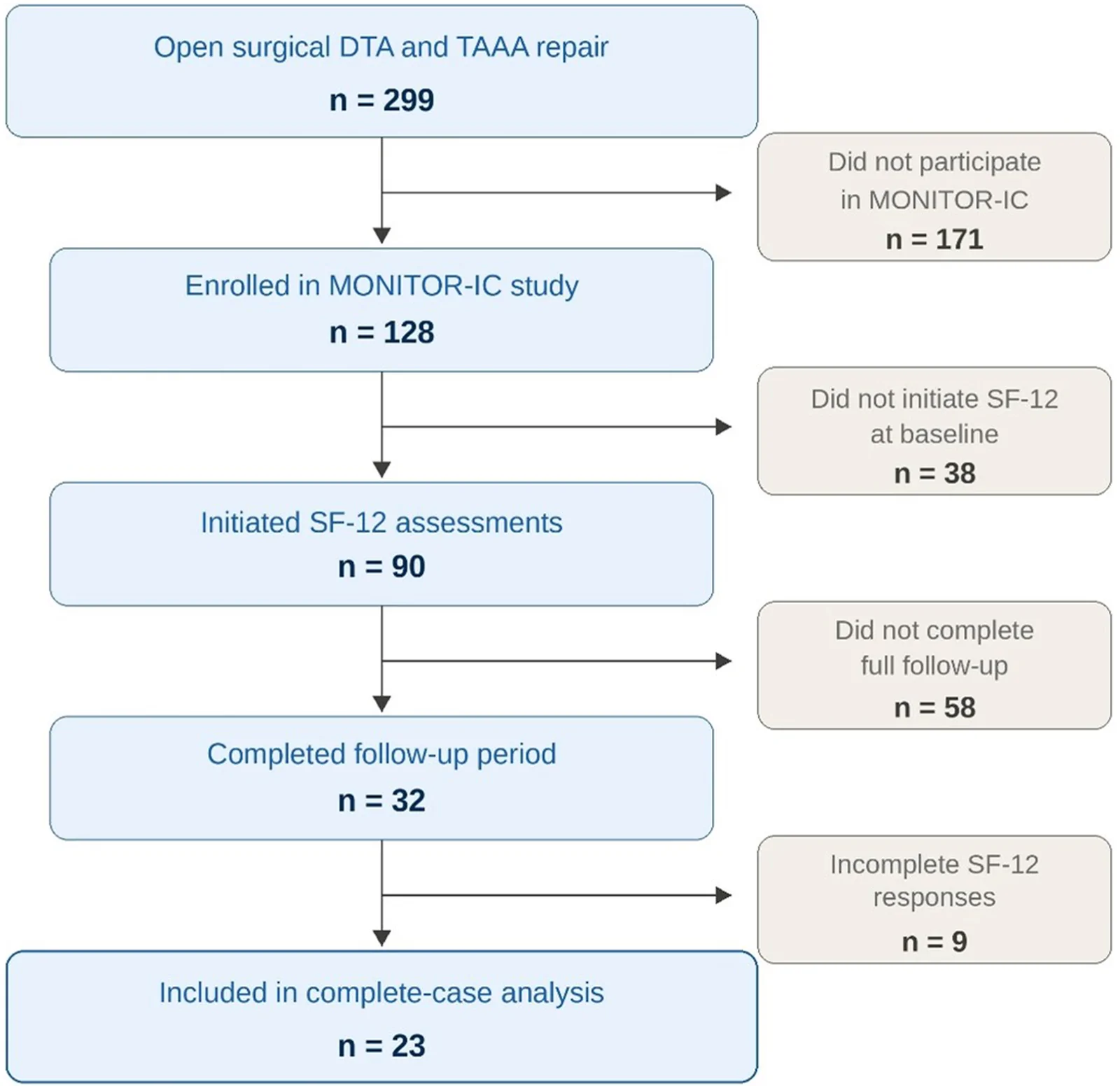
Flow diagram of study participants. *DTA*, Descending thoracic aortic aneurysm; *TAAA*, thoracoabdominal aortic aneurysm; *SF-12*, 12-item Short-Form Health Survey.

**Table 1:** Baseline demographic and clinical characteristics of included patients

Characteristic	Frequency (n = 128)
Age, y	65.5 (57.3–70.8)
Male sex	73 (57%)
Body mass index, kg/m^2^	26.8 ± 4.4
Acute presentation	13 (10.2%)
Preoperative unstable condition	1 (0.8%)
Aneurysm characteristics	
Descending thoracic aneurysm	40 (31.3%)
Thoracoabdominal aneurysm	88 (68.8%)
Postdissection aneurysm	62 (48.4%)
Crawford classification	
Type I	30 (34.1%)
Type II	20 (22.7%)
Type III	11 (12.5%)
Type IV	23 (26.1%)
Type V	4 (4.5%)
Comorbidities	
Coronary artery disease	19 (14.8%)
Pulmonary disease	23 (18.0%)
Pulmonary hypertension	1 (0.8%)
Peripheral artery disease	26 (20.3%)
Preoperative neurological deficit	18 (14.1%)
Connective tissue disease	19 (14.8%)
Diabetes mellitus-type 2	4 (3.1%)
Previous surgery	
Previous aortic surgery	67 (52.3%)
Previous cardiac surgery	12 (9.4%)
Previous lung surgery	2 (1.6%)

Continuous variables are presented as mean ± standard deviation (normality) or median with interquartile range (nonnormality). Categorical variables are reported as number of patients (n) with percentages.

Thoraco-phreno-laparotomy was the most common surgical approach (68.8%, ie, all TAAA patients), and extracorporeal circulation was used in 98.4% of cases. Intraoperative complications occurred in 5.5%. Postoperatively, reintubation occurred in 11.7% and pulmonary complications in 37.5%. Acute kidney injury developed in 27.3%, with 2.3% requiring dialysis and 1.6% remaining dialysis-dependent at discharge. Reoperation was performed in 7.8%, and neurological complications occurred in 20.3%; 88.5% of the neurological complications resolved. There was a long-term mortality of 7.8% (4.2 per 100 patient-years) (Table 2).

**Table 2:** Operative and postoperative details of included patients

Characteristic	Frequency or value
Surgical approach	
Thoracotomy	40 (31.2%)
Thoraco-phreno-laparotomy	88 (68.8%)
Extracorporeal circulation	
ECC used	126 (98.4%)
Conventional ECC	15 (11.7%)
Partial ECC (femoral)	23 (18.0%)
Left heart bypass	88 (68.8%)
Organ protection strategies	
Deep hypothermia	8 (6.3%)
Circulatory arrest	8 (6.3%)
Selective antegrade cerebral perfusion	13 (10.2%)
Cardioplegia	15 (11.8%)
Nephroplegia	53 (41.4%)
Operative times	
ECC time, minutes	118.33 ± 60.3
Circulatory arrest temperature, °C	20.0 (18.5-20.0)
Circulatory arrest time, minutes	36.0 ± 16.2
Aortic clamp time, minutes	84.0 (54.0-120.0)
Intraoperative outcomes	
Intraoperative complications	7 (5.5%)
Postoperative respiratory outcomes	
Reintubation	15 (11.7%)
Pulmonary complication	48 (37.5%)
Postoperative renal outcomes	
Acute kidney injury	35 (27.3%)
Postoperative dialysis	3 (2.3%) (2 at discharge)
Postoperative neurological outcomes	
Neurological disorder	26 (20.3%)
Paraparesis	4 (3.1%) (3 recovered)
Paralysis	1 (0.8%) (0 recovered)
CVA	4 (3.1%) (all recovered)
Vocal cord paralysis	14 (10.9%) (13 recovered)
Other	5 (3.9%) (all recovered)
Reintervention	
In-hospital reoperation	10 (7.8%)
Mortality	
30-day mortality	2 (1.6%)
Long-term mortality	10 (7.8%)

Continuous variables are presented as mean ± standard deviation (normality) or median with interquartile range (nonnormality). Categorical variables are reported as number of patients (n) with percentages. *ECC*, Extracorporeal circulation; *CVA*, cerebrovascular accident.

### Health-Related QoL Outcome

#### Short form-12

Estimated marginal means for PCS-12 were 46.0 (95% CI, 44.1-48.0) preoperatively, 39.7 (95% CI, 37.6-41.7) at 3 months, 42.2 (95% CI, 40.2-44.2) at 12 months, and 41.0 (95% CI, 38.1-43.9) at 24 months (Figure 2). A significant overall difference was observed (χ^2^(3), = 36.9, *P* < .001), with significantly lower scores at all postoperative time points versus baseline (all *P* < .01). No significant differences were found between postoperative time points (all *P* > .07). The estimated MCID was 5.0 (consistent with literature^19^).

**Figure 2:**
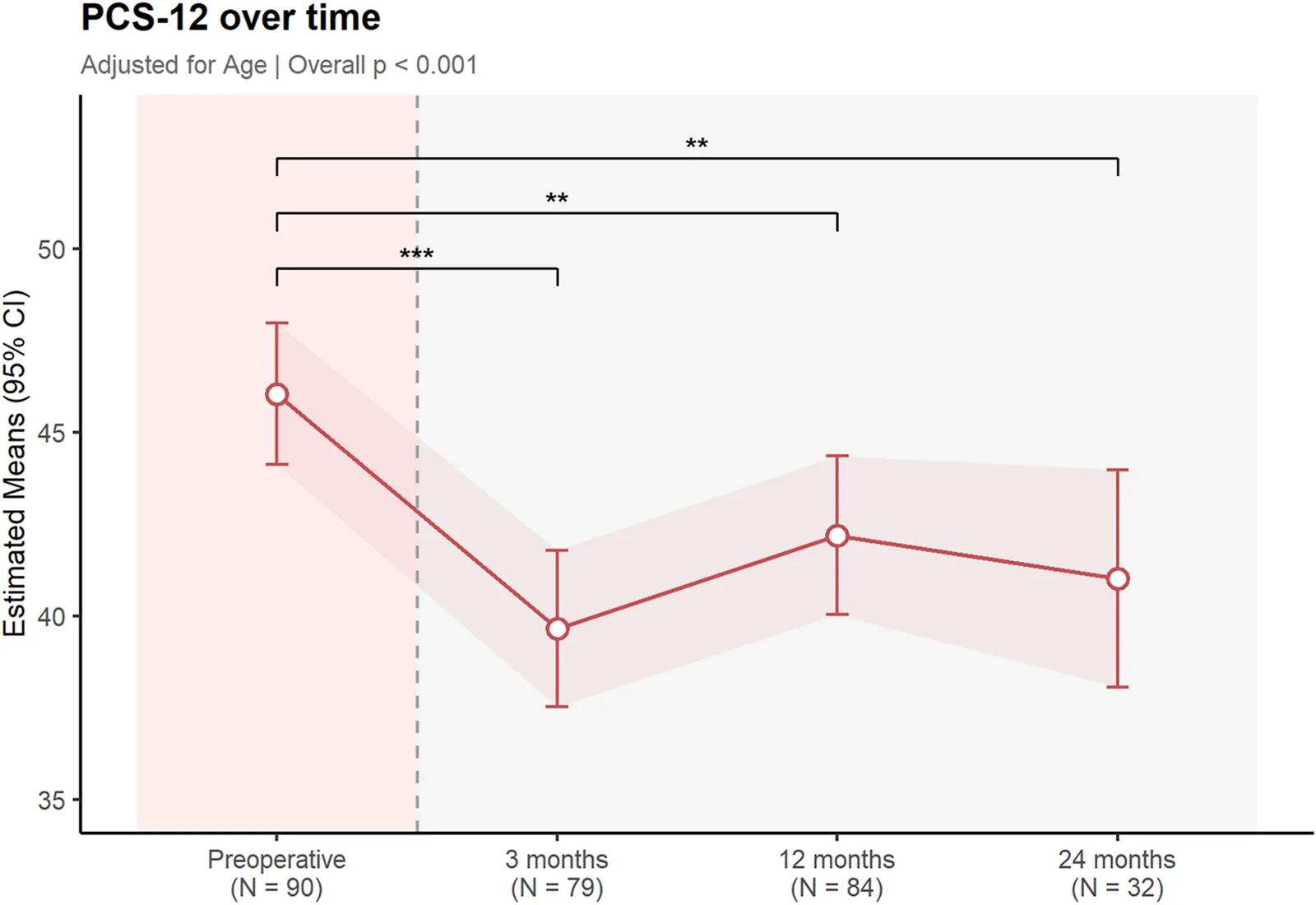
Estimated marginal means of PCS-12 scores at the preoperative assessment and during postoperative follow-up (including 95% CIs). Significant pairwise comparisons are indicated in the figure. *PCS-12*, Short Form-12 physical component summary. *∗∗P* < .010; *∗∗∗P* < .001.

A significant overall difference of MCS-12 scores was also observed (χ^2^(3) = 10.5, *P* = .01) (Figure 3). Estimated marginal means were 47.4 (95% CI, 44.9-49.8) preoperatively, 44.3 (95% CI, 41.8-46.9) at 3 months, 49.2 (95% CI, 46.7-51.7) at 12 months, and 48.4 (95% CI, 44.6-52.2) at 24 months. The only significant pairwise difference was between 3 and 12 months (*P* = .01). The estimated MCID for MCS was 5.3, consistent with literature (5.2).^19^

**Figure 3:**
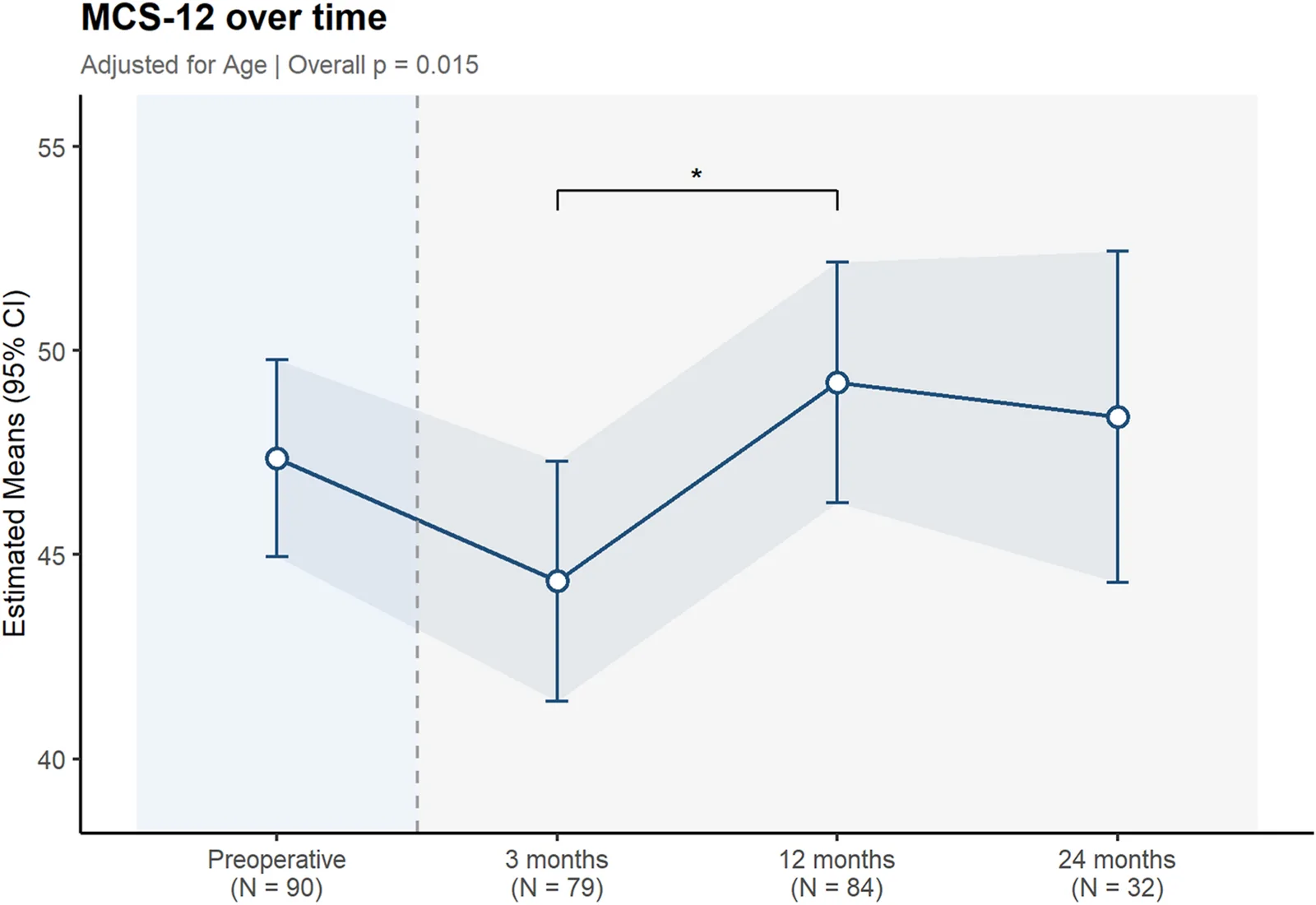
Scores at the preoperative assessment and during postoperative follow-up (including 95% CIs). Significant pairwise comparisons are indicated in the figure. *MCS-12*, Short Form-12 mental component summary. *∗P* < .050.

The complete-case analysis (n = 23, Figure 1) showed a similar temporal pattern with higher *P* values. A significant overall time effect remained for PCS-12 (χ^2^(3) = 10.71, *P* = .01), with only the baseline-to-3-month comparison significant (*P* = .010). No significant effect was found for MCS-12 (χ^2^(3) = 5.92, *P* = .12). No baseline variables were independently associated with PCS-12 or MCS-12 trajectories, except for a borderline significant association between BMI and MCS-12 (Tables E2 and E3). Within the subgroup analyses, the only significant difference in PCS scores was found between patients with complicated versus uncomplicated postoperative courses (38.8 ± 9.5 vs 43.4 ± 6.58 [uncorrected *P* = .022, corrected *P* = .087]). There were no other significant differences (Table E4).

#### Patient-reported outcome measures

After adjustment for multiple testing, statistically significant changes over time were observed for fatigue (CIS, *P* < .01), frailty (CFS, *P* = .02), cognitive failure (CFQ, *P* = .02), and anxiety symptoms (HADS-A, *P* = .04). No significant changes were found for HRQoL (EQ-5D-5L, *P* = .12), depressive symptoms (HADS-D, *P* = .19), or posttraumatic stress symptoms (IES-6, *P* = .26). Graphical representations of all secondary outcomes are provided in Figures E1-Figure E2, Figure E3.

Fatigue (CIS) increased significantly at 3 months compared with baseline (*P* < .01), followed by a significant decrease toward baseline levels at 12 and 24 months (3 vs 12 months, *P* = .01; 3 vs 24 months, *P* = .01). Frailty (CFS) showed a similar pattern, with an increase at 3 months compared to baseline (*P* = .01), followed by a decrease over time, without additional significant pairwise differences. Cognitive failure (CFQ) worsened over time, with higher scores at 12 and 24 months compared with baseline (*P* = .01 and *P* = .02, respectively). Conversely, anxiety symptoms (HADS-A) improved over time, with a significant reduction in anxiety between baseline and 24 months (*P* = .01) (Table 3).

**Table 3:** Estimated marginal means with 95% CIs for PROMs derived from linear mixed models and corrected *P* values (Holm-Bonferroni)

PROM	Preoperative	3 months	12 months	24 months	Overall (corrected) *P* value
PCS-12	46.0 (44.1-48.0)	39.7 (37.6-41.7)	42.2 (40.2-44.2)	41.0 (38.1-43.9)	**<.01**
MCS-12	47.4 (44.9-49.8)	44.3 (41.8-46.9)	49.2 (46.7-51.7)	48.4 (44.6-52.2)	**.01**
NLEQ5D5L	0.8 (0.7-0.8)	0.7 (0.7-0.8)	0.8 (0.7-0.8)	0.7 (0.7-0.8)	.12
CFS	2.8 (2.6-3.0)	3.2 (3.0-3.4)	2.9 (2.7-3.1)	3.0 (2.8-3.3)	**.02**
CIS	30.1 (27.8-32.3)	35.3 (32.9-37.6)	31.0 (28.5-33.4)	31.2 (28.5-33.9)	**<.01**
HADS-A	5.7 (5.0-6.5)	4.8 (4.1-5.6)	4.8 (4.0-5.6)	4.3 (3.4-5.2)	**.04**
HADS-D	5.1 (4.3-5.8)	5.7 (5.0-6.5)	4.8 (4.0-5.6)	5.0 (4.1-5.9)	.19
IES-R	NA	0.6 (0.5-0.7)	0.5 (0.4-0.6)	0.5 (0.4-0.7)	.26
CFQ	20.9 (18.0-23.7)	23.5 (18.0-26.2)	24.7 (21.8-27.6)	24.9 (21.8-28.0)	**.02**

Statistically significant *P* values (*P* < .05) are shown in bold. *PROM*, Patient-reported outcome measures; *PCS-12*, physical component summary (Short Form-12); *MCS-12*, mental component summary (Short Form-12); *NLEQ5D5L*, Dutch EuroQol 5 dimensions 5 levels questionnaire; *CFS*, Clinical Frailty Scale; *CIS*, checklist individual strength; *HADS-A*, Hospital Anxiety and Depression Scale, Anxiety; *HADS-D*, Hospital Anxiety and Depression Scale, Depression; *IES-R*, impact of event scale - revised; *CFQ*, Cognitive Failure Questionnaire.

## Discussion

This study prospectively evaluated multiple HRQoL domains in patients undergoing open DTA and TAAA repair. Both physical and mental HRQoL was below population norms at baseline (<50)^20^ with a further decline in the early postoperative phase. However, the longitudinal recovery trajectory differed. Physical HRQoL declined at three months, and showed only partial recovery without returning to preoperative levels, suggesting a lasting functional impairment despite technical success. This pattern is consistent with previous research.^5^^,^^9^^,^^21^ Additionally, the PCS-12 decline exceeded the calculated MCID, indicating that patients perceive this deterioration as clinically meaningful. This trajectory contrasts with the general cardiac surgery population, in which physical health status typically recovers more rapidly due to the less invasive access (ie, sternotomy vs thoraco-phreno-laparotomy).^22^

Mental HRQoL scores showed an initial decline followed by recovery, ultimately exceeding baseline levels. Pairwise comparisons were nonsignificant and effect sizes modest (MCS-12 difference < MCID), possibly due to limited statistical power from missing data. The divergence between persistent physical impairment and improving mental HRQoL is noteworthy. This may reflect relief of perceived aneurysm-related risk or a response shift phenomenon, in which patients adjust their expectations following major surgery. It is consistent with the significant reduction in HADS-A scores, previous literature, and chronic thoracic aneurysm literature.^21^^,^^23^ Recovery, and possible improvement of the mental health status, is consistent with general cardiac surgery literature as well.^22^

Secondary physical outcomes (eg, fatigue and frailty) followed a similar trajectory with varying effect sizes, thereby supporting the observed effect of longer-term physical impairment after open surgical repair. Secondary mental outcomes were more heterogeneous, with baseline scores generally within normal ranges. HADS-D mirrored the MCS-12 pattern without reaching significance, while HADS-A decreased significantly, consistent with reduced perceived threat. Self-reported cognitive failure increased significantly but remained within normal limits. The sustained rise in cognitive complaints contrasts with the typically transient deficits reported after aortic surgery, though existing literature remains heterogeneous.^24^ IES-6 scores were stable, indicating no substantial trauma-related distress.

Complete-case analyses preserved overall patterns with reduced statistical strength, consistent with limited power from missing data and patients who were not included in the MONITOR-IC study (Figure 1). No baseline variables were associated with PCS-12 or MCS-12 trajectories, though previous literature has identified female sex, smoking, older age, prior vascular interventions, and comorbidities such as chronic obstructive pulmonary disease as relevant risk factors.^5^^,^^21^ However, the current analyses were likely underpowered to detect such associations. Furthermore, no difference was observed between the DTA and TAAA groups, which is likely because two-thirds of the cohort consisted of TAAA patients, and these findings predominantly reflect that subgroup. Other subgroup analyses were not feasible due to the limited number of included patients and statistical power.

### Strengths and Limitations

The prospective longitudinal design with preoperative baseline and follow-up to 24 months provides a comprehensive overview of HRQoL trajectories after open DTA and TAAA repair. Inclusion of a real-world cohort of both elective and acute patients enhances external validity, and the use of multiple PROMs enabled extensive assessment of physical, mental, and cognitive domains.

Missing data at longer follow-up and the voluntary nature of the study may have introduced selection bias and limited statistical power (Figure 1), though linear mixed-effects models and subgroup analyses were used to mitigate this. The single-center design limits generalizability but ensured consistency in surgical technique and perioperative management. Multiple testing increases type I error risk, which was addressed through appropriate corrections. Retrospective assessment of preoperative HRQoL and the use of proxy respondents may have introduced bias, though this influence is likely limited given the consistency of longitudinal patterns, the low proportion of patients (∼10%) who used proxies, and previous literature confirming the validity of proxy assessments.^25^

### Clinical Implications

Despite varying effect sizes, the consistent pattern of reduced physical functioning suggests that preoperative counseling and clinical decision-making should explicitly address the possible deterioration with incomplete recovery. Given lower-than-normal baseline mental health scores, preoperative psychological support and education may help address anxiety and disease-related burden. Additionally, based on insights into preoperative functioning domains, a more patient-tailored treatment and postoperative recovery plan could be developed. Finally, this information should inform the shared decision-making process, as recommended in the guidelines,^1^ to improve recovery perception and postoperative impairment.

### Future Research

Future research should investigate the relationship between physical and mental recovery, as both similar and distinct patterns were observed. Patient-centered and qualitative research could offer further insight into how these outcomes interact. Furthermore, it could provide information regarding the true perceived impact of the differences in HRQoL found in this study. Although multiple PROMs were used, none fully align with the specific needs of DTA and TAAA patients. Developing a disease-specific PROM would improve assessment homogeneity, as recommended by a previous systematic review.^5^ Additionally, comparing PROMs with objective functioning scores would complement these findings. Studies with more complete follow-up are needed to strengthen the evidence base. Finally, future research should explore whether perioperative management strategies can optimize both physical and mental HRQoL after DTA and TAAA surgery.

## Conclusions

This study provides insights across multiple domains of physical, mental, and cognitive functioning in the perioperative period of invasive open surgical DTA or TAAA repair. In this cohort of patients undergoing open DTA or TAAA repair, physical HRQoL declined both statistically significantly and clinically meaningfully (exceeding MCID) after surgery with only partial recovery. Secondary physical outcomes followed a similar trajectory. Mental HRQoL showed a transient decline followed by recovery with a trend toward exceeding preoperative levels, with more heterogeneous patterns across secondary mental outcomes. No baseline characteristics significantly influenced physical or mental trajectories. Overall, open DTA and TAAA repair imposes a lasting physical burden whereas mental health appears to recover and possibly improve, highlighting the importance of integrating patient-centered outcomes into clinical decision-making and perioperative care (Figure 4).

**Figure 4:**
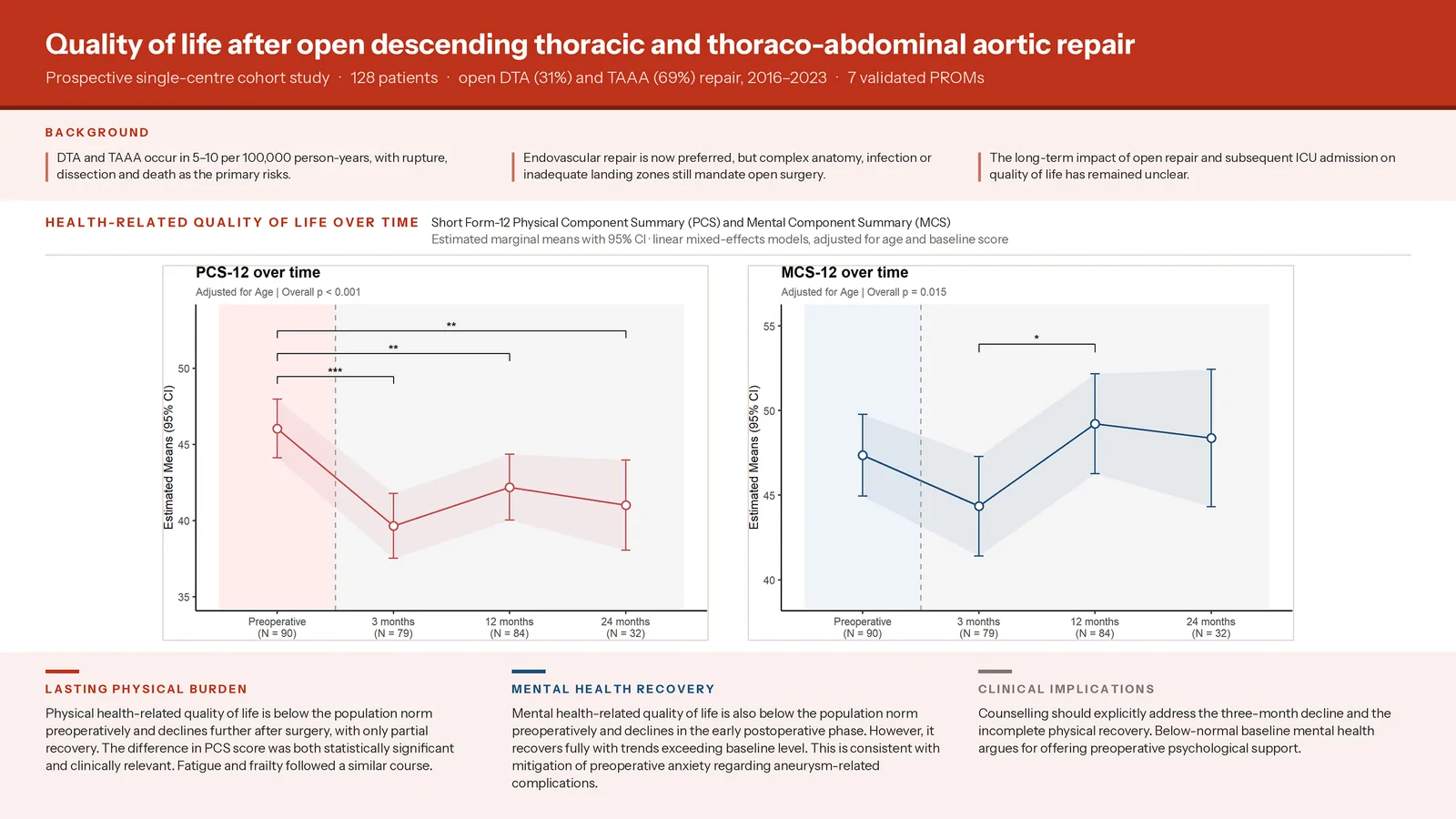
Graphical abstract summarizing methods and results of quality-of-life outcomes after open descending thoracic and thoracoabdominal aortic repair. ∗*P* < .050; *∗∗P* < .010; *∗∗∗P* < .001. *PCS-12*, Short Form-12 physical component summary; *MCS-12*, Short Form-12 mental component summary; *DTA*, descending thoracic aortic aneurysms; *TAAA*, thoracoabdominal aortic aneurysms.

## Conflict of Interest Statement

The authors reported no conflicts of interest.

The *Journal* policy requires editors and reviewers to disclose conflicts of interest and to decline handling or reviewing manuscripts for which they may have a conflict of interest. The editors and reviewers of this article have no conflicts of interest.

## Figures and Tables

**Table E1:** Characteristics of the included patient reported outcome measures (PROMS) with score ranges and reference values or cut-off values per score

PROM	Subject	Characteristics	Score range	Cut-off values
SF-12 11^E1^	HRQoL	Eight health concepts devided in PCS and MCS	0-100 per component	NA
EQ-5D-5 L^E2^	HRQoL	5 domains (mobility, self-care, usual activities, pain, anxiety/depression) with 5 point likert scale and visual analogue scale (EQ-VAS 0-100)	−0.45-1	NA
CFS^E3^	Frailty	9 point frailty scale (Very fit to Terminally ill)	1-9	5
CIS-8^E4^	Fatigue	8-item questionnaire scored on a 7-point Likert scale	8-56	27
HADS^E5^	Anxiety and Depression	14-item questionnaire with separate subscales for anxiety and depression	0-21 per component	8 per component
IES-6^E6^	Post-traumatic stress	6 items investigating symptoms of intrusion, avoidance, and hyperarousal	0-1 (normally 0-4)	1.75
CFQ-14^E7^	Cognition	14 items evaluating frequency of cognitive lapses in attention, memory, and action	0-100	43

*SF-12*, Short form; *NA*, not applicable; *CFS*, clinical frailty scale; *CIS*, checklist individual strength; *HADS*, hospital anxiety & depression scale; *IES*, impact of event scale; *CFQ*, cognitive failure questionnaire.

**Figure E1:**
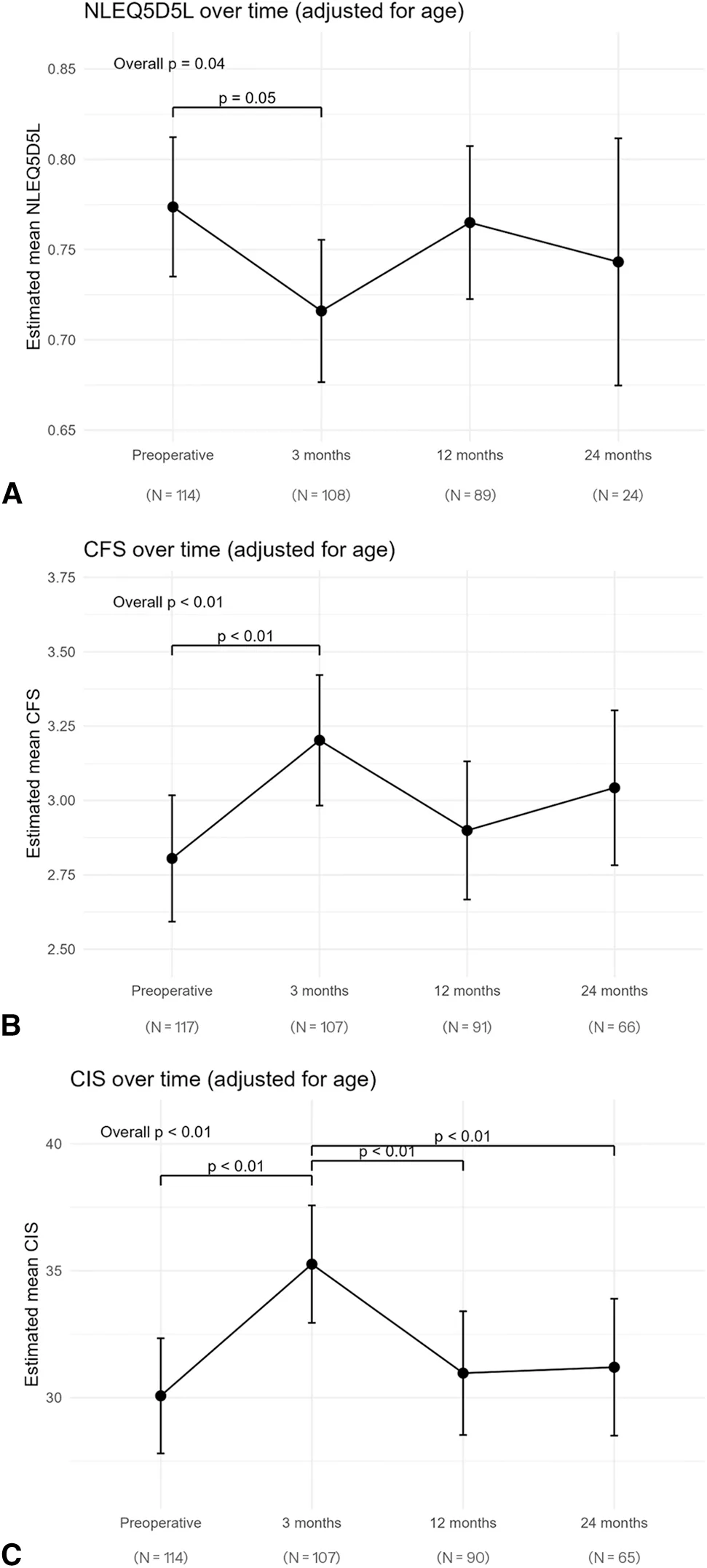
Estimated marginal means of the secondary physical HRQoL (A: Dutch EuroQol 5 dimensions 5 levels questionnaire, B: Clinical Frailty Scale, C: Checklist Individual Strength) outcomes derived from the primary linear mixed-effects model. Error bars represent 95% confidence intervals.

**Figure E2:**
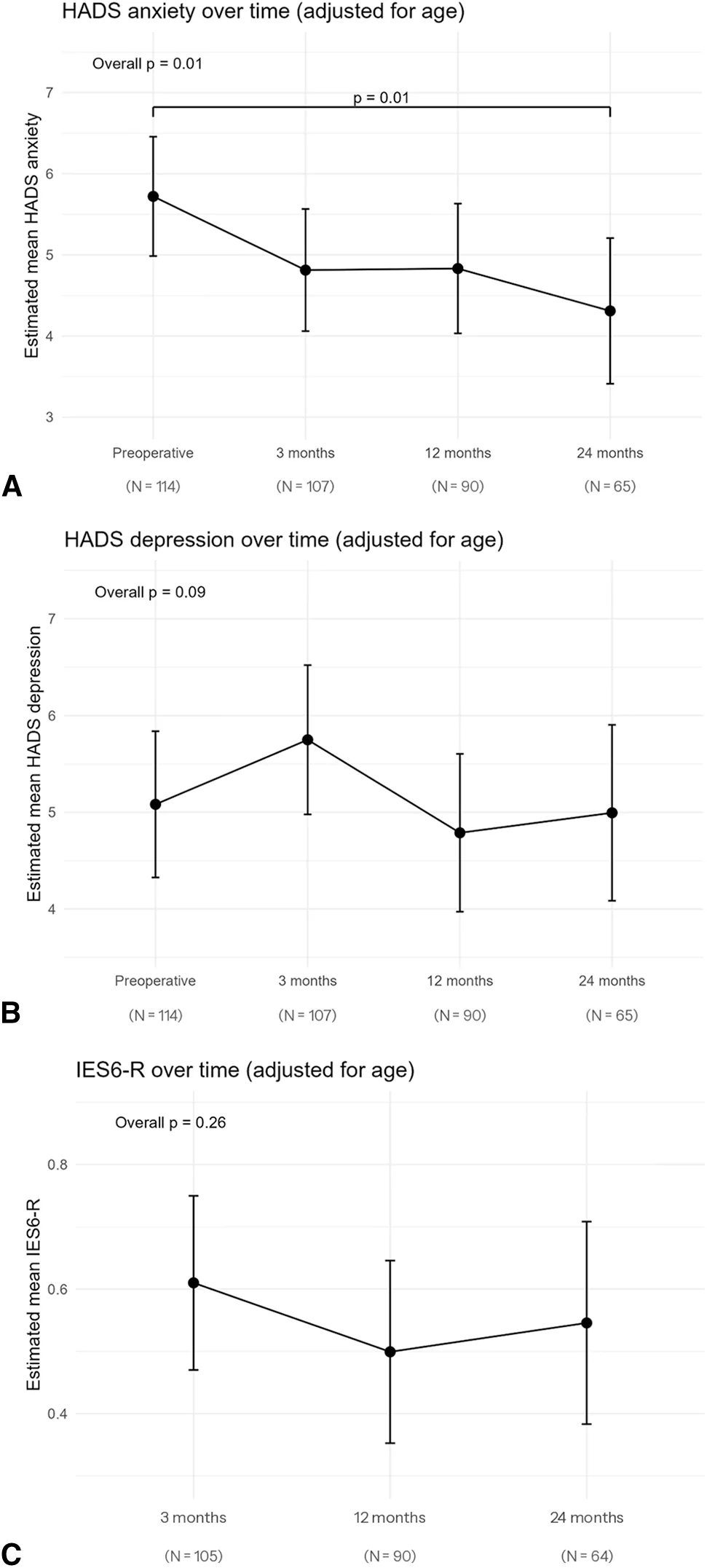
Estimated marginal means of the secondary mental HRQoL (A & B: Hospital Anxiety and Depression Scale, C: Impact of Event Scale) outcomes derived the primary linear mixed-effects model. Error bars represent 95% confidence intervals.

**Figure E3:**
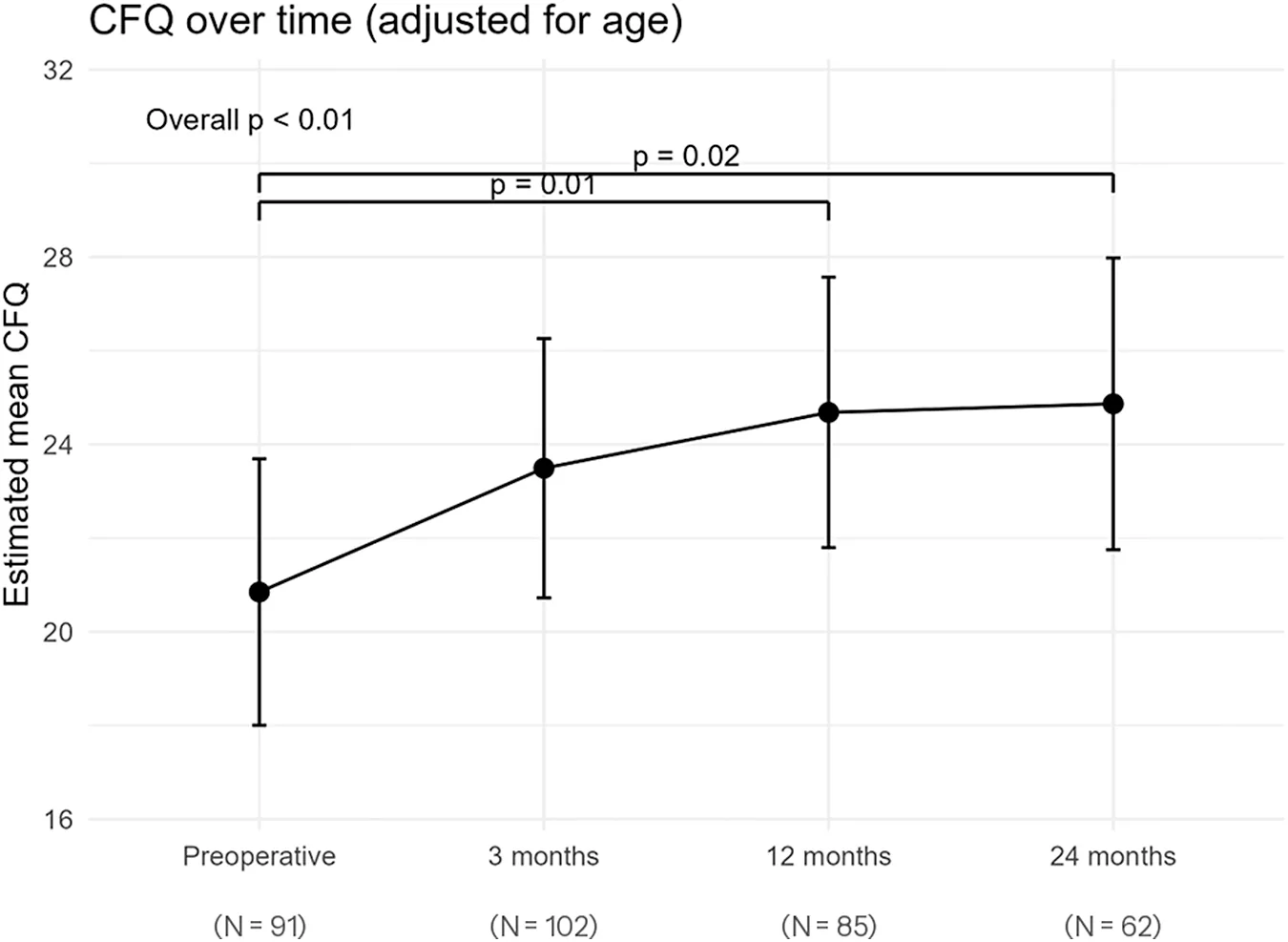
Estimated marginal means of the Cognitive Failure Questionnaire outcomes derived the primary linear mixed-effects model. Error bars represent 95% confidence intervals.

**Table E2:** Univariable linear mixed-effects model results for the association between baseline covariables and longitudinal physical and mental health-related quality of life

Variable	Estimate	Std. error	df	*t* value	*P* value
PCS-12					
Age	−0.01	0.06	103.77	−0.22	.83
Gender (female)	−0.49	1.56	103.34	−0.31	.75
BMI	−0.11	0.18	97.43	−0.65	.52
Coronary artery disease	0.84	2.14	102.40	0.39	.70
Pulmonary disease	0.83	1.93	102.73	0.43	.67
Peripheral artery disease	−1.12	1.81	99.51	−0.62	.54
Previous aortic surgery	−0.23	1.54	103.17	−0.15	.88
Previous cardiac surgery	−2.82	2.48	105.27	−1.14	.26
DM type 2	0.55	3.90	83.60	0.14	.89
Acute presentation	−2.55	2.41	104.07	−1.06	.29
MCS-12					
Age	0.02	0.08	100.54	0.21	.83
Gender (female)	1.01	1.82	98.58	0.56	.58
BMI	−0.33	0.20	91.66	−1.63	.11
Coronary artery disease	2.71	2.49	98.97	1.09	.28
Pulmonary disease	0.77	2.26	98.46	0.34	.74
Peripheral artery disease	1.16	2.10	92.64	0.55	.58
Previous aortic surgery	−2.50	1.78	98.91	−1.41	.16
Previous cardiac surgery	1.62	2.92	101.64	0.55	.58
DM type 2	−3.36	4.46	76.12	−0.75	.45
Acute presentation	−3.36	2.81	98.53	−1.20	.23

Estimates represent fixed effects (β) with corresponding standard errors (SE), degrees of freedom (df), *t*-values, and *P*-values. *BMI*, Body mass index; *DM*, diabetes mellitus.

**Table E3:** Multivariable linear mixed-effects model results for the association between baseline covariables and longitudinal physical and mental health-related quality of life

Variable	Estimate	Std. error	df	*t* value	*P* value
PCS-12					
Age	−0.01	0.07	93.5	−0.16	.87
Gender (female)	−0.35	1.82	96.26	−0.19	.85
BMI	−0.12	0.21	93.66	−0.55	.59
Coronary artery disease	2.30	2.75	96.02	0.84	.41
Pulmonary disease	0.81	2.17	95.31	0.37	.71
Peripheral artery disease	−1.48	2.01	95.04	−0.73	.46
Previous aortic surgery	0.15	1.80	94.93	0.08	.93
Previous cardiac surgery	−4.29	2.95	98.18	−1.45	.15
DM type 2	0.30	4.54	80.41	0.07	.95
Acute presentation	−3.21	2.60	96.22	−1.24	.22
MCS-12					
Age	−0.01	0.08	83.68	−0.16	.87
Gender (female)	0.63	2.02	86.97	0.31	.76
BMI	−0.46	0.23	83.94	−1.98	.05
Coronary artery disease	0.64	3.07	86.74	0.21	.84
Pulmonary disease	−1.24	2.42	86.07	−0.51	.61
Peripheral artery disease	1.54	2.24	84.41	0.69	.49
Previous aortic surgery	−2.16	2.00	85.23	−1.08	.28
Previous cardiac surgery	1.46	3.30	90.18	0.44	.66
DM type 2	−6.07	4.93	68.00	−1.23	.22
Acute presentation	−3.88	2.89	85.67	−1.34	.18

Estimates represent fixed effects (β) with corresponding standard errors (SE), degrees of freedom (df), *t*-values, and *P*-values. *BMI*, Body mass index; *DM*, diabetes mellitus.

**Table E4:** Differences in PCS 12 and MCS 12 scores between subgroups: Descending thoracic aneurysm (DTA) versus Thoraco-abdominal aneurysm (TAAA), Elective presentation versus Acute presentation, Complicated versus Uncomplicated postoperative course derived by Welch *t*-test or Mann–Whitney *U* tests depending on normality (Shapiro–Wilk) with uncorrected *p* value and Holm-Bonferroni corrected *p* value

DTA vs TAAA							
PROM	Time point	Score DTA	n DTA	Score TAAA	n TAAA	*P* value uncorrected	*P* value corrected
PCS12	Preoperative	48.6 [38.5-55.5]	30	46.9 [40.4-54.8]	60	.811	1.000
PCS12	3 mo	42.5 ± 8.3	24	38.94 ± 9.2	55	.093	.370
PCS12	12 mo	43.6 ± 8.5	25	41.0 [36.5-49.7]	59	.389	1.000
PCS12	24 mo	42.5 ± 10.1	13	40.1 ± 8.7	19	.489	1.000
MCS12	Preoperative	52.4 [38.6-56.6]	30	48.6 [40.1-56.9]	60	.925	1.000
MCS12	3 mo	48.0 ± 12.0	24	45.2 [35.3-55.0]	55	.124	.452
MCS12	12 mo	55.9 [47.9-58.3]	25	51.9 [41.3-56.5]	59	.113	.452
MCS12	24 mo	50.7 ± 12.2	13	48.7 ± 13.1	19	.653	1.000

**Elective vs acute presentation**							
**PROM**	**Time point**	**Score elective**	**n elective**	**Score acute**	**n acute**	***P* value uncorrected**	***P* value corrected**
PCS12	Preoperative	47.2 [40.4-55.5]	80	44.1 ± 9.0	10	.326	1.000
PCS12	3 mo	40.2 ± 9.0	71	38.2 ± 9.9	8	.599	1.000
PCS12	12 mo	42.05 [37.0-50.5]	75	40.5 ± 6.5	9	.418	1.000
PCS12	24 mo	41.6 ± 9.8	26	38.9 ± 6.4	6	.427	1.000
MCS12	Preoperative	50.0 [39.9-56.8]	80	47.5 ± 12.6	10	.913	1.000
MCS12	3 mo	46.64 [36.3-56.0]	71	46.9 ± 15.5	8	.165	.659
MCS12	12 mo	52.9 [45.2-57.3]	75	43.9 ± 16.3	9	1.000	1.000
MCS12	24 mo	50.8 ± 11.6	26	50.7 ± 11.6	6	.364	1.000

**Complicated vs uncomplicated postoperative course**							
**PROM**	**Time point**	**Score complicated**	**n complicated**	**Score uncomplicated**	**n uncomplicated**	***P* value uncorrected**	***P* value corrected**
PCS12	3 mo	38.8 ± 9.5	58	43.4 ± 6.58	21	.022	.087
PCS12	12 mo	39.1 [36.4-48.9]	62	44.9 ± 8.2	22	.111	.334
PCS12	24 mo	40.3 ± 9.4	27	45.3 ± 7.7	5	.241	.481
MCS12	3 mo	45.7 [33.5-55.9]	58	47.1 ± 9.4	21	.538	1.000
MCS12	12 mo	52.9 [43.8-57.8]	62	52.1 [42.7-56.1]	22	.586	1.000
MCS12	24 mo	51.3 [43.9-58.4]	27	49.6 ± 13.5	5	1.000	1.000

Values are presented as mean ± standard deviation or median [Interquartile ranges].

## References

[1] Isselbacher E.M., Preventza O., Hamilton Black Iii J. (2022). 2022 ACC/AHA guideline for the diagnosis and management of aortic disease: a report of the American heart Association/American College of Cardiology Joint Committee on clinical practice guidelines. J Am Coll Cardiol.

[2] Hasami N.A., Smulders M., Verkroost M.W.A. (2025). Open surgery of the descending thoracic aorta in the endovascular era: a single centre experience. Eur J Vasc Endovasc Surg.

[3] Lau C., Ram E., Harik L. (2025). Long-term clinical outcomes of 1020 open repairs of descending thoracic and thoracoabdominal aortic aneurysms. J Thorac Cardiovasc Surg.

[4] Frankort J., Doukas P., Krabbe J. (2026). Outcomes following contemporary open thoracoabdominal aortic aneurysm extent I repair-a cross-border study. Eur J Cardiothorac Surg.

[5] Lampridou S., Saghdaoui L.B., Bicknell C., Kumlien C., Lear R. (2024). Health related quality of life following intervention for thoracoabdominal aortic aneurysm: a systematic review and narrative synthesis. Ann Vasc Surg.

[6] Hasami N.A., Smulders M., Verkroost M.W.A. (2025). Contemporary outcomes of open thoraco-abdominal aortic repair: a single-centre experience. Interdiscip Cardiovasc Thorac Surg.

[7] Stoecker J.B., Wang G.J. (2021). Epidemiology of thoracoabdominal aortic aneurysms. Semin Vasc Surg.

[8] Ishaque S., Karnon J., Chen G., Nair R., Salter A.B. (2019). A systematic review of randomised controlled trials evaluating the use of patient-reported outcome measures (PROMs). Qual Life Res.

[9] Jarral O.A., Kidher E., Patel V.M., Nguyen B., Pepper J., Athanasiou T. (2016). Quality of life after intervention on the thoracic aorta. Eur J Cardio-Thoracic Surg.

[10] Geense W., Zegers M., Vermeulen H., van den Boogaard M., van der Hoeven J. (2017). MONITOR-IC study, a mixed methods prospective multicentre controlled cohort study assessing 5-year outcomes of ICU survivors and related healthcare costs: a study protocol. BMJ Open.

[11] Gandek B., Ware J.E., Aaronson N.K. (1998). Cross-validation of item selection and scoring for the SF-12 Health Survey in nine countries: results from the IQOLA Project. International Quality of Life Assessment. J Clin Epidemiol.

[12] Herdman M., Gudex C., Lloyd A. (2011). Development and preliminary testing of the new five-level version of EQ-5D (EQ-5D-5L). Qual Life Res.

[13] Rockwood K., Song X., MacKnight C. (2005). A global clinical measure of fitness and frailty in elderly people. Cmaj.

[14] Vercoulen J.H., Swanink C.M., Fennis J.F., Galama J.M., van der Meer J.W., Bleijenberg G. (1994). Dimensional assessment of chronic fatigue syndrome. J Psychosom Res.

[15] Zigmond A.S., Snaith R.P. (1983). The hospital anxiety and depression scale. Acta Psychiatr Scand.

[16] Weiss D.S. (2007). Cross-cultural assessment of psychological trauma and PTSD.

[17] Wassenaar A., de Reus J., Donders A.R.T. (2018). Development and validation of an abbreviated questionnaire to easily measure cognitive failure in ICU survivors: a multicenter study. Crit Care Med.

[18] Norman G.R., Sloan J.A., Wyrwich K.W. (2003). Interpretation of changes in health-related quality of life: the remarkable universality of half a standard deviation. Med Care.

[19] Grand N., Bouchet J.B., Zufferey P. (2018). Quality of life after cardiac operations based on the minimal clinically important difference concept. The Ann Thorac Surg.

[20] Ware J.E., Sherbourne C.D. (1992). The MOS 36-item short-form health survey (SF-36). I. Conceptual framework and item selection. Med Care.

[21] Sharples L.D., Anagnostopoulou V., Pouncey A.L. (2024). Longitudinal health-related quality of life in people with thoracic aortic aneurysms. Br J Surg.

[22] Raidou V., Mitete K., Kourek C. (2024). Quality of life and functional capacity in patients after cardiac surgery intensive care unit. World J Cardiol.

[23] Thijssen C.G.E., Dekker S., Bons L.R. (2020). Health-related quality of life and lived experiences in males and females with thoracic aortic disease and their partners. Open Heart.

[24] Benson R.A., Ozdemir B.A., Matthews D., Loftus I.M. (2017). A systematic review of postoperative cognitive decline following open and endovascular aortic aneurysm surgery. Ann R Coll Surg Engl.

[25] Hofhuis J., Hautvast J.L.A., Schrijvers A.J.P., Bakker J. (2003). Quality of life on admission to the intensive care: can we query the relatives?. Intensive Care Med.

